# Behavior of Lebanese Pediatricians regarding Children’s Oral Health

**DOI:** 10.5005/jp-journals-10005-1469

**Published:** 2017-02-27

**Authors:** Balsam Noueiri, Nahla Nassif, Riad Bacho

**Affiliations:** 1 Associate Professor, Department of Pediatric Dentistry, School of Dentistry Lebanese University, Beirut, Lebanon; 2 Assistant Professor, Department of Pediatric Dentistry, School of Dentistry Lebanese University, Beirut, Lebanon; 3 Assistant Professor, Department of Pediatric Dentistry, School of Dentistry Lebanese University, Beirut, Lebanon

**Keywords:** Behavior, Children, Medical visit, Oral health, Pediatricians, Teeth.

## Abstract

**Aim:**

The aim of the study is to evaluate the behavior of Lebanese pediatricians regarding children’s oral health.

**Materials and methods:**

A cross sectional study including 100 Lebanese pediatricians was performed. They answered 11 questions. Three variables were taken into consideration: The number of years in practice, the place and the type of practice.

**Results:**

The answers were tabulated according to the latter three subdivisions. 97.7% of pediatricians who have been practicing for more than 5years reported inquiring about whether a child is taking a milk/juice bottle overnight as compared to 76.9% of pediatricians who have been practicing for less than five years. The majority of pediatricians working in cities/big villages (98.9%) and 76.9% in small villages do look for cavities during oral examination.

**Discussion:**

Independently of the years in practice, the majority of pediatricians look for cavities and check the mucosa/ tongue during the oral examination. The results regarding dental examination revealed a significant difference between the pediatricians working in cities/big villages (98.9%) and those in small villages (76.9%). Regarding the frequent diagnosis of ECC, there is also a significant difference between pediatricians working in cities/ big villages (74.7%) and those working in small villages (46.2%).

**Conclusion:**

The behavior of the Lebanese pediatricians regarding children’s oral health is not satisfactory. In their daily practice, pediatricians need to have more interaction with pediatric dentists and should take into consideration the AAP and AAPD recommendations.

**How to cite this article:** Noueiri B, Nassif N, Bacho R. Behavior of Lebanese Pediatricians regarding Children’s Oral Health. Int J Clin Pediatr Dent 2017;10(4):379-383.

## INTRODUCTION

Oral health is an integral aspect of the general health that can influence both quality-of-life and mortality of individuals.^1^ Dental caries is one of the most prevalent oral conditions, wherein, according to the World Oral Health Report, around 60 to 90% of school-aged children are affected by the condition in developed countries.^2^ The global increase in dental caries prevalence is found affecting children as well as adults and primary as well as permanent teeth. This increase in dental caries signals a pending public health crisis.^3^

According to the American Academy of Pediatric Dentistry,^4^ a delay in initiating dental hygiene practices by parents until after the eruption of several teeth, overnight bottle/breastfeeding, high intake of sweetened beverages or carbohydrates, delaying the first dental visit until after the first year of age, and delaying oral hygiene practices are all factors that have been attributed to the increased risk of early childhood caries (ECCs).

A study conducted in the United States by Caspary et al,^5^ on residents in the Department of Pediatrics showed that most of them consider oral health screening as one of their responsibilities. Furthermore, in a study conducted in Turkey, the majority of pediatricians were more likely to believe that check-up visits should include oral health assessments.^6^ A study conducted on Canadian family doctors and pediatricians revealed that majority of both groups believe that their practice plays a significant role in the children’s oral health.^7^

Inadequate knowledge has been observed in several domains, such as recommending of the use of fluoridate toothpastes for the prevention of dental caries.^8,9^

Furthermore, there has been a discrepancy between the latter positive reported attitude of pediatricians toward oral health assessment and their actual practices. According to Lewis et al,^10^ more than 90% of the pediatricians said that they should examine their patients’ teeth and educate families about preventive oral health. However, only 54% of pediatricians reported examining the teeth of more than half of their 0- to 3-year-old patients.^6^ Although many pediatricians reported inspecting the oral cavity of children,^7,8,10,11^ fewer pediatricians were likely to inquire about bottle feeding during night,^6^ educate parents about significance of tooth brushing, and recommend the first dental visit to be done before 1 year of age.^11,12^

Knowledge deficiency and improper practice have been attributed to the lack of education and training in oral health assessments during both medical school education and residency training.^13^ Several studies showed that pediatricians have reported that they did not receive adequate information during their medical formation.^5,6,10,11^

Pediatricians are not only the first health providers, but they also play an important role in the early identification and prevention of oral health problems.

This specific role can be either positive if the pediatricians are applying their dental knowledge in their practice, or negative if they do not provide the parents with all the necessary recommendations and advice.

For the first time in Lebanon, a study was conducted to assess the behavior of Lebanese pediatricians toward children’s oral health issues, in their daily practice.

The aim of this study is to evaluate their behavior regarding children’s oral health. In addition to the above-mentioned purpose, the second objective of this study is to assess whether the pediatricians’ behaviors differed in terms of their years of experience (more than or less than 5 years), place of practice (city/big village *vs* small village), and type of practice (private with or without academic affiliation defined by teaching in local universities), by answering a questionnaire inspired from similar studies,^8,12^ adapted to the context, pretested, and modified accordingly.

## MATERIALS AND METHODS

A cross-sectional approach was utilized to conduct this study. Our population of interest was Lebanese pediatrician members of the Lebanese Society of Pediatricians. They were selected from five governorates, namely, Beirut, Mount Lebanon, North Lebanon, South Lebanon, and Bekaa. The number of doctors from each governor-ate was proportional to the total number of pediatricians practicing in this governorate. A total of 100 pediatricians were interviewed at their workplaces: 22 from Beirut, 43 from Mount Lebanon, 11 from North Lebanon, 15 from South Lebanon, and 8 from Bekaa.

The survey was composed of 11 questions divided into two sections: general information and behavior regarding oral health. The general information included the following variables: the years of practice, which is divided into less than and more than 5 years, the place of practice, which is divided into city/big village and small village and, finally, the type of practice, which is divided into either private practice or having an academic affiliation. Pediatricians’ behavior was measured using the following variables: age at which they recommend to stop overnight bottle/breastfeeding, the referral age for the first dental visit, the recommended age to start teeth cleaning, and the amount of fluorinated toothpaste they recommend. Looking for dental caries and oral lesions as well as overnight intake of milk or juice and diagnosis of ECC were also measured as variables. All questionnaires were completely filled, with no answers were missing.

Data were analyzed using Statistical Package for the Social Sciences version 18. Categorical data were analyzed using chi-squared test, whereas continuous variables were analyzed using t-test.

## RESULTS

A total of 100 Lebanese pediatricians returned the questionnaire duly completed. The answers were tabulated according to three independent variables: years in practice, type of practice, and practice area.

About 87% of pediatricians had more than 5 years of experience and 13% had less than 5 years; 87% worked in cities/big villages and 13% in small villages. In relation to the type of practice, 63% of pediatricians practiced exclusively in the private sector and 37% had a private practice and an academic affiliation (Table 1).

The results were analyzed and discussed in Tables 2 to 4.

## DISCUSSION

Recognition of the profound deleterious effects of oral problems on children’s quality-of-life has led to a growing interest in the role of pediatricians in early diagnosis in oral pathology.

This current study was conducted on a sample volume of 100 Lebanese pediatricians representing proportionally the five governorates. About 87% have been practicing since more than 5 years and they are basically considered well experienced. The remaining 13% have less than 5 years of practice. Only 37% of them have a private practice with an academic affiliation and possessing scientific and medical updates.

The majority of pediatricians work in cities or big villages (87%). This fact can be attributed to the presence of a highly dense population in the cities, which can lead to higher number of patients compared with small villages. This was noticed too by Probst et al^14^ and Rosenthal et al.^15^

**Table 1:** **Table 1:** General information

*Parameters*		*Percentage of pediatricians (%)*	
Years of practice: More than 5 years		87	
Less than 5 years		13	
Practice area: City/big village		87	
Small village		13	
Type of practice: Private practice only		63	
With academic affiliation		37	

**Table 2:** **Table 2:** Pediatricians’ behavior characteristics by years of practice

				*Years in practice*					
*Questions*				*More than 5 years*		*Less than 5 years*		*p-value*	
Do you look for cavities during oral examination		Yes		97.7%		84.6%		0.081	
Do you examine a child’s mucosa/tongue		Yes		98.9%		100.0%		0.870	
Do you inquire whether a child is taking milk/juice from a		Yes		97.7%		76.9%		0.015	
bottle overnight									
How often do you diagnose ECC		Frequently		75.9%		38.5%		0.002	
		Rarely/never		24.1%		61.6%			
At what age do you recommend to stop overnight feeding		Before 9 months		51.7%		7.7%		0.001	
		Before 18 months		40.2%		53.8%			
		After 18 months		8.0%		38.5%			
At what age do you recommend to start teeth cleaning		1st tooth		51.7%		46.2%		0.469	
		Several teeth		48.3%		53.8%			
At what age do you refer the child for a first dental visit		1 year		10.3%		15.4%		0.074	
		3 years old		83.9%		61.5%			
		5 years old		5.7%		23.1%			
What amount of fluorinated toothpaste do you recommend		None		9.2%		30.8%		0.005	
		Pea size		39.1%		61.5%			
		Along the length of		51.7%		7.7%			
		the toothbrush							

**Table 3:** **Table 3:** Pediatricians’ behavior characteristics by practice area

				*Practice area*					
*Questions*				*City/big village*		*Small village*		*p-value*	
Do you look for cavities during oral examination		Yes		98.9%		76.9%		0.007	
Do you examine a child’s mucosa/tongue		Yes		98.9%		100.0%		0.870	
Do you inquire whether a child is taking milk/juice from		Yes		97.7%		76.9%		0.015	
a bottle overnight									
How often do you diagnose ECC		Frequently		74.7%		46.2%		0	
		Rarely/never		25.3%		53.9%			
At what age do you recommend to stop overnight feeding		Before 9 months		51.7%		7.7%		0.005	
		Before 18 months		39.1%		61.5%			
		After 18 months		9.2%		30.8%			
At what age do you recommend to start teeth cleaning		1st tooth		54.0%		30.8%		0.102	
		Several teeth		46.0%		69.2%			
At what age do you refer the child for a first dental visit		1 year		11.5%		7.7%		0.005	
		3 years old		83.9%		61.5%			
		5 years old		4.6%		30.8%			
What amount of fluorinated toothpaste do you recommend		None		13.5%		0%		0.016	
		Pea size		37.1%		81.8%			
		Along the length of		49.4%		18.2%			
		the toothbrush							

**Table 4:** **Table 4:** Pediatricians’ behavior characteristics by type of practice

				*Practice area*					
*Questions*				*Private practice*		*Academic affiliation*		*p-value*	
Do you look for cavities during oral examination		Yes		95.5%		100.0%		0.623	
Do you examine a child’s mucosa/tongue		Yes		100.0%		90.9%		0.110	
Do you inquire whether a child is taking milk/juice from		Yes		96.6%		81.8%		0.092	
a bottle overnight									
How often do you diagnose ECC		Frequently		70.8%		72.7%		0.825	
		Rarely/never		29.2%		27.3%			
At what age do you recommend to stop overnight feeding		Before 9 months		49.4%		18.2%		0.016	
		Before 18 months		37.1%		81.8%			
		After 18 months		13.5%		0%			
At what age do you recommend to start teeth cleaning		1st tooth		52.8%		36.4%		0.240	
		Several teeth		47.2		63.6%			
At what age do you refer the child for a first dental visit		1 year		11.2%		9.1%		0.970	
		3 years old		80.9%		81.8%			
		5 years old		7.9%		9.1%			
What amount of fluorinated toothpaste do you recommend		None		8%		38.5%			
		Pea size		40.2%		53.8%			
		Along the length of		51.7%		0.001%			
		the toothbrush							

The results showed that, independently of the years in practice, the majority of pediatricians look for cavities and check the mucosa/tongue during the oral examination. It indicates their concern about the oral cavity as well as the general child’s health (Table 2).

On the contrary, the results regarding dental examination revealed a significant difference between the pediatricians working in cities/big villages and those working in small villages (Table 3).

Interestingly, in small villages, 100% of pediatricians check the oral soft tissues; however, only 76.9% of them look for cavities. This can reveal that the pediatricians do not have a relatively high degree of knowledge regarding oral health, explaining their self-reported behavior.

Other studies showed more consistent results between the number of physicians looking for both cavities and performing an oral exam.^8,16^ In 2007, Sandalli et al^6^ showed that 38% of pediatricians frequently examine children’s teeth for dental problems.

Regarding the overnight intake of milk/juice from a bottle, there were several significant differences found between the responses of more experienced pediatricians and the less experienced ones (Table 2) and between pediatricians working in cities/big villages and those in small villages (Table 3). According to Sandalli et al,^6^ 22.3% of pediatricians question whether the child sleeps with a baby bottle.

The assessment of the behavior of pediatricians regarding the diagnosis of ECC frequently showed that 75.9% of pediatricians with more than 5 years of experience and 38.5% with less than 5 years were able to diagnose ECC (Table 2). Similarly, we noticed a significant difference between the pediatricians working in cities/big villages at 74.7% and 46.2% as working in small villages (p 0.000) (Table 3). Kagihara et al^17^ mentioned that dental caries is the most common chronic disease of childhood, occurring 5 times as frequently as asthma and seven times more commonly than hay fever. This is the reason the authors insist on the significant role of a primary care health provider in the prevention of ECC. Krol^18^ said that, optimally, a physician sees a child 8 times in the first year and around 12 times by age of 3. These regular visits would allow for early assessment of the child’s oral health and referral for dental problems and prevention.

The principal factor implicated in ECC is the overnight intake of milk/juice. Independently of the number of years in practice, the practice area, and the type of practice, the results of the present study showed that most of pediatricians do not apply the American Academy of Pediatrics (AAP) and the American Academy of Pediatric Dentistry (AAPD) recommendations about weaning the child before the age of 9 months.^4,19^

The highest percentages of right answers was 51% by pediatricians working for more than 5 years (Table 2), and by those working in cities/big villages (Table 3). However, only 18.2% of pediatricians with an academic affiliation were able to answer correctly (Table 4). About 91.6% of the respondents knew that putting a baby in bed with a bottle of milk/juice is harmful. However, for Retna Kumari et al,^20^ the percentage was 63.5% and 72.6% for Shivaprakash et al.^21^

Pediatricians are able to educate parents about basic preventive dental care and counsel them long before dentists. They see the child 8 times in the first year and around 12 times by age of 3.^18^

In the present study, the highest percentage of pediatricians who recommend cleaning the first erupted tooth was 54% (Table 3). But, many problems in the oral cavity that are common in childhood can be prevented with the pediatrician’s guidance.^22^

Concerning the first dental visit, the highest percentage of right answers is 15.4, 11.5, and 11.2% respectively, presented in Tables 2 to 4. Those dramatic results show that the majority of the Lebanese pediatricians are not following the new AAP and AAPD guidelines. The first examination is recommended at the time of the eruption of the first tooth and no later than 2 months of age.^23,24^

The answers about the recommended amount of fluorinated toothpaste were equally divided between those who recommend “applying along the length of the bristles” and those who recommend a pea size or none. So, the Lebanese pediatrician’s tendency is to use an excess amount of toothpaste. It reflects on their lack of knowledge and this could be due to the impact of commercials.

To minimize the risk of fluorosis in children, while maximizing the caries-prevention benefit for all age groups, the appropriate amount of fluoride toothpaste should be used by all children regardless of age.^25^

## CONCLUSION

This finding elicits the following concern: the behavior of the Lebanese pediatricians regarding children’s oral health is not satisfactory. This fact leads to the following suggestions:

- Effective application of their dental knowledge is needed for good oral health.
- Collaboration between pediatric dentists and pediatricians is recommended not only through education programs, but also by promoting oral examinations of the child during his first year of life.
- Continuous updating on the AAPD and AAP guidelines is required.
- Applying the recommendations of the AAPD and AAP must become a routine in their daily practice.
- Informing parents about the importance of child’s oral hygiene should be adhered to in every child’s general consultation.

## References

[1] Filstrup SL, Briskie D, da Fonseca M, Lawrence L, Wandera A, Inglehart MR (2003). Early childhood caries and quality of life: child and parent perspectives.. Pediatr Dent.

[2] Petersen PE (2003). The World Oral Health Report 2003: continuous improvement of oral health in the 21st century - the approach of the WHO Global Oral Health Programme.. Community Dent Oral Epidemiol.

[3] Bagramian RA, Garcia-Godoy F, Volpe AR (2009). The global increase in dental caries. A pending public health crisis.. Am J Dent.

[4] (2014). American Academy of Pediatric Dentistry. Guideline on infant oral health care.. Reference Manual.

[5] Caspary G, Krol DM, Boulter S, Keels MA, Romano-Clarke G (2008). Perceptions of oral health training and attitudes toward performing oral health screenings among graduating pediatric residents.. Pediatrics.

[6] Sandalli N, Kuvvetli SS, Cildir SK, Ergeneli S (2007). “The pediatricians” role in the oral health of children.. OHDMBSC.

[7] Prakash P, Lawrence HP, Harvey BJ, Mclsaac WJ, Limeback H, Leake JL (2006). Early childhood caries and infant oral health: Paediatricians’ and family physicians’ knowledge, practices and training.. Paediatr Child Health.

[8] Soares IMV, Silva, da Silva AMR, Moura LFA, de Lima MDM, Netto OBS, de Moura MS (2013). Conduct of pediatricians in relation to the oral health of children.. Rev Odontol UNESP.

[9] Balaban R, Aguiar CM, da Silva Araujo AC, dias Filho EB (2012). Knowledge of paediatricians regarding child oral health.. Int J Paediatr Dent.

[10] Lewis CW, Boulter S, Keels MA, Krol DM, Mouradian WE, O’Connor KG, Quinonez RB (2009). Oral health and pediatricians: results of a national survey.. Acad Pediatr.

[11] Indira MD, Dhull KS, Nandlal B (2015). Knowledge, attitude and practice toward infant oral healthcare among the pediatricians of Mysore: a questionnaire survey.. Int J Clin Pediatr Dent.

[12] Bhat PK, Aruna C, Badiyani BK, Alle R (2014). Knowledge and attitude on infant oral health among graduating medical students in Bangalore City, India.. JIMSA.

[13] Olatosi OO, Iwuala SO, Ojewola RW, Chukwudifu N, Oredugba FA, Sote EO (2016). Undergraduate medical students’ knowledge and attitude on early childhood caries and infant oral health.. J Pediatr Dent.

[14] Probst JC, Moore CG, Baxley EG, Lammie JJ (2002). Rural-urban differences in visits to primary care physicians.. Fam Med.

[15] Rosenthal MB, Zaslavsky A, Newhouse JP (2005). The geographic distribution of physicians revisite.. Health Serv Res.

[16] Gebhard K, Bernstein B An assessment of Connecticut pediatricians’ recommendations regarding oral health care in young children. Available from:. www.cbe.uchc.edu/education/selectives/pdfs/paper.../sel-gebhardt.pdf..

[17] Kagihara LE, Niederhauser VP, Stark M (2009). Assessment, management, and prevention of early childhood caries.. Am Acad Nurse Pract.

[18] Krol DM (2004). Educating pediatricians on children’s oral health: past, present, and future.. Pediatrics.

[19] (2014). Americain Academy of Pediatricians. Updates schedule of screening and assessment for well-child visit..

[20] Retna Kumari N, Sheela S, Sarada PN (2006). Knowledge and attitude on infant oral health among graduating students in Kerela.. J Indian Soc Pedod Prev Dent.

[21] Shivaprakash PK, Elango I, Baweja DK, Noorani HH (2009). The state of infant oral healthcare knowledge and awareness: disparity among parents and healthcare professionals.. J Indian Soc Pedod Prev Dent.

[22] Herndon JB, Tomar SL, Lossius MN, Catalanotto FA (2010). Preventive oral health care in early childhood: knowledge, confidence and practices of pediatricians and family physicians in Florida.. J Pediatr.

[23] (2014). American Academy of Pediatrics. Recommendations for preventive pediatric healthcare.. Pediatrics.

[24] (2015). American Academy of Pediatric Dentistry. Guideline on periodicity of examination, preventive dental services, anticipatory guidance/counseling, and oral treatment for infants, children, and adolescents.. Pediatr Dent.

[25] Wright JT, Hanson N, Ristic H, Whall CW, Estrich CG, Zentz RR (2014). Fluoride toothpaste efficacy and safety in children younger than 6 years: a systematic review.. J Am Dent Assoc.

